# Efficacy of radial extracorporeal shock wave therapy in hereditary spastic paraplegia: A case report

**DOI:** 10.1097/MD.0000000000041921

**Published:** 2025-08-08

**Authors:** Akira Shutoh, Risa Harada, Ryoga Kashima, Wataru Saho, Ryo Yoshikawa, Yoshitada Sakai

**Affiliations:** a Division of Rehabilitation Medicine, Kobe University Hospital, Kobe, Japan; b Division of Rehabilitation Medicine, Kobe University Graduate School of Medicine, Kobe, Japan.

**Keywords:** extracorporeal shock wave therapy, hereditary spastic paraplegia, spasticity

## Abstract

**Rationale:**

Hereditary spastic paraplegia (HSP), a rare neurodegenerative disorder in Japan, is characterized by insidiously progressive lower extremity spasticity and muscle weakness. Current management is primarily symptomatic, including physical therapy and spasticity modulation with botulinum toxin or intrathecal baclofen. Recent studies have suggested that extracorporeal shock wave therapy (ESWT) may be effective for treating spasticity in patients recovering from stroke. We report a case exploring the use of radial ESWT (rESWT) combined with physiotherapy in a patient with HSP.

**Patient concerns:**

A 31-year-old man diagnosed with HSP in his 20s had been receiving physical therapy and orthotics but experienced progressive bilateral lower extremity spasticity, leading to deterioration in gait function and mobility.

**Diagnoses:**

Progressive HSP with exacerbated bilateral lower extremity spasticity manifesting as impaired gait efficiency and reduced functional mobility.

**Interventions:**

The patient was treated with rESWT focusing bilaterally on the calf muscles.

**Outcomes:**

Posttreatment, the patient’s visual analog scale score for ease of walking improved significantly from 40 to 85, reflecting enhanced walking ability. The timed up and go test results improved from 15.08 to 12.59 seconds and 11.51 to 10.43 seconds at comfortable and maximum speeds, respectively. The 10-meter walk test time decreased from 10.48 to 7.24 seconds, and the step count improved from 20 to 14. Despite the modified Ashworth scale scores and dorsiflexion measurements showing minimal changes, no adverse effects were observed.

**Lessons:**

This case report provides preliminary evidence that rESWT combined with physical therapy may improve gait disturbances, as well as the ease of walking and ambulatory ability, associated with spasticity in HSP. However, due to the limitations of a single-case study and the potential influence of confounding factors, further research – including randomized, placebo-controlled trials – is necessary to confirm its efficacy and determine optimal treatment parameters.

## 1. Introduction

Hereditary spastic paraplegia (HSP) is a group of neurodegenerative disorders characterized by slowly progressive lower limb spasticity and muscle weakness.^[1]^ Its prevalence in Japan is low, at approximately 0.2 per 100,000 people.^[2]^

No effective treatment has been established for HSP. Current symptomatic treatments include physical therapy (gait, joint range of motion, and muscle strength training) and spasticity management using interventions such as local injections of botulinum toxin or prolonged intrathecal baclofen administration.^[3]^

Recently, extracorporeal shock wave therapy (ESWT) has been reported as an effective novel treatment for spasticity in patients recovering from stroke or hypoxic encephalopathy.^[4,5]^ In this study, we combined physiotherapy with radial ESWT (rESWT) to treat lower limb spastic paraplegia associated with HSP. Improvements in walking speed, stride length, and the patient’s visual analog scale (VAS) scores for ease of walking were observed.

This study conformed to all CARE guidelines and reports the required information accordingly.

## 2. Case presentation

The patient was a 31-year-old male without perinatal or significant motor development abnormalities, although his father and grandfather had gait disturbances. He first noticed difficulty running in his teens. In his 20s, he was admitted to the Neurology department for examination and was diagnosed with HSP at the hospital. He is currently undergoing genetic testing at the Japan Spastic Paraplegia Research Consortium. On his first visit to the rehabilitation department, inpatient physical therapy was initiated along with the prescription of a pair of shoehorn braces (SHB) for both lower limbs. However, the SHB broke after a few months due to the progressive spastic paraplegia and his weight, prompting the use of a short-leg orthosis with a carbon fiber-reinforced plastic 3-layer ankle-foot orthotic device with a posterior strut (SAWAMURA Orthotics and Orthotics Service Co., Hyogo, Japan).

**Figure 1.**
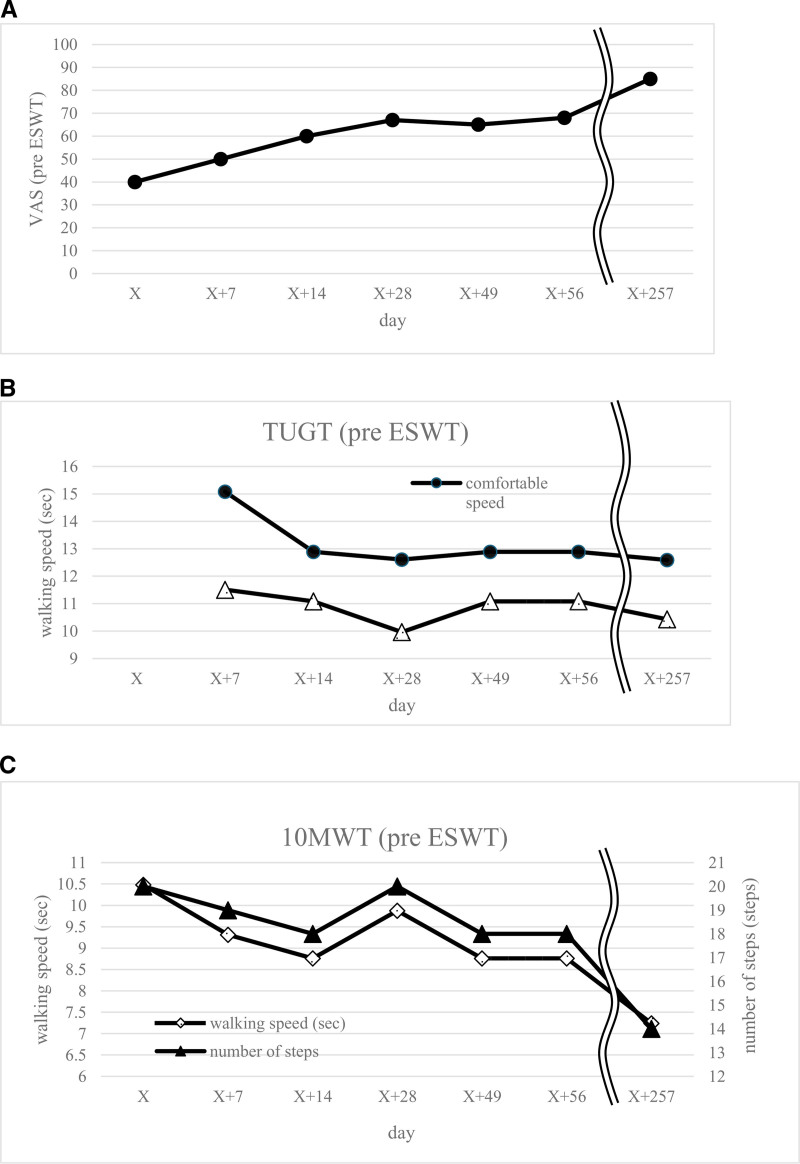
Clinical course of the symptoms and effects of rESWT. Prolonged rESWT improved the ease of walking and the ability to walk. rESWT was performed at the hospital on day X, X + 7, X + 14, X + 28, X + 49, and X + 56. Thereafter, rESWT was administered every 2 weeks at a rehabilitation clinic. rESWT = radial extracorporeal shockwave therapy.

The patient initially experienced satisfactory walking with the braces, but his lower limb spastic paralysis gradually worsened, resulting in increased walking difficulty. Although botulinum toxin therapy was recommended, the patient was reluctant to receive injections. Consequently, rESWT was administered to address the spasticity, and improvements in walking speed and stride length were noted. The irradiation energy, which is highest in the superficial layers of the skin, attenuates with depth. In Japan, rESWT is covered by insurance for refractory plantar tendonitis. rESWT was performed using a PHYSIO SHOCKMASTER device (SAKAI Medical Co., Ltd. Tokyo, Japan) and was administered once every 1 to 3 weeks, focusing on the bilateral triceps surae muscles. The intensity (2.2 bar), frequency (12 Hz), and number of shots (1500 per session) were set according to previous systematic reviews.^[6]^ Figure 1 illustrates that rESWT was performed on day X, X + 7, X + 14, X + 28, X + 49, and X + 56. After 6 sessions of rESWT at the hospital, the patient was transferred to a rehabilitation clinic where he continued receiving rESWT and physical therapy every 2 weeks. To date, the patient remains under this combined treatment regimen. Botulinum toxin was not administered.

Clinical assessments included the VAS for ease of walking, the modified Ashworth scale (MAS) of both ankle joints, bilateral dorsiflexion with knee joint extension (DKE) and with knee joint flexion (DKF) range of motion, the timed up and go test (TUGT), and 10-meter walk test (10 MWT). The VAS evaluated the subjective ease of walking, while the TUGT assessed performance at the comfortable and maximum speeds. The occurrence of side effects, such as bleeding and pain, was also monitored. Written informed consent was obtained from the patient prior to inclusion in the study and for publication of this case. The confidentiality of the participant was maintained, and all data were anonymized. The study adhered to ethical standards and guidelines for research involving human subjects.

## 3. Results

The VAS score improved from 40 before the first rESWT session to 85 posttreatment. TUGT at comfortable speed improved from 15.08 to 12.59 seconds, and at maximum speed from 11.51 to 10.43 seconds. The 10 MWT improved from 10.48 to 7.24 seconds, and the number of steps reduced from 20 to 14 steps (Fig. 1). Notably, the MAS score remained unchanged at 3. Meanwhile, DKE improved slightly (right:–45° to –35°; left:–60° to –55°), and DKF improved slightly on the right (–45° to –35°) but showed no change on the left (–45° to –45°). No adverse effects were observed. Although the patient had a history of mild intellectual disability and complicated depression, these conditions were not severe enough to affect treatment adherence or the reliability of the evaluation, and rESWT was performed safely without the need for anesthesia.

## 4. Discussion

The clinical course of this case highlights 2 important findings. First, rESWT may have contributed to improvements in gait performance and VAS scores despite the advanced HSP. Second, while rESWT has been explored as a possible intervention for spasticity, further studies are needed to determine its efficacy, particularly in cases where botulinum toxin therapy is either not indicated or refused.

An important point to clarify is the relationship between spasticity as measured by the MAS and functional improvement. In this case, although the MAS score – a standard indicator of muscle tone and spasticity – remained unchanged, significant functional improvements were observed in gait performance (as evidenced by improvements in the TUGT and 10 MWT). This suggests that rESWT may not dramatically alter baseline muscle tone as quantified by the MAS, but it might improve the viscoelastic properties of muscles and tendons or enhance local circulation.

Although the mechanism behind the improvement in spasticity remains unresolved, mechanical stimulation of the neuromuscular junction presumably induces the release of nitric oxide, which is involved in vasodilation and anti-inflammatory effects, thereby increasing angiogenesis in the muscles and tendons, improving muscle viscoelasticity, and promoting tissue repair. Mechanical stimulation of the neuromuscular junction reportedly denatures the acetylcholine receptors and causes temporary nerve motor impairment; however, this was based on an experiment in rats.^[7]^

Such changes could result in better functional performance without a marked change in spasticity scores. In other words, while traditional spasticity metrics did not reflect significant change, factors such as improved muscle elasticity or enhanced blood flow may have contributed to the observed improvements in walking speed and stride length.

Regarding the duration of effect, previous reports in children with cerebral palsy suggest that the benefits of ESWT on spasticity might last for approximately 1 to 3 months before diminishing. In our case, the beneficial effects were observed to persist at least 2 weeks posttreatment. However, further long-term follow-up is necessary to fully understand the duration of rESWT’s benefits in HSP.

The comparison between rESWT and botulinum toxin therapy also merits discussion. Although the SPASTOX study indicated that botulinum toxin improved muscle tone without significantly enhancing motor function in HSP,^[8]^ the efficacy of botulinum toxin in HSP remains uncertain due to interpatient variability.

Whether botulinum toxin therapy or ESWT or a combination of the 2 is more effective has not been elucidated. A comparison of ESWT and botulinum toxin therapy for reducing spasticity in patients with cerebral palsy showed they were both useful.^[9]^ In the treatment of spasticity in cases of stroke, the combination of botulinum toxin therapy and ESWT has been reported to be more effective in decreasing the MAS score, increasing the joint range of motion and reducing pain in spastic limbs.^[10]^ However, no comparative studies in patients with HSP have been reported.

In our case, the functional improvements with rESWT – combined with its noninvasive administration – suggest that it might offer advantages when botulinum toxin therapy is not feasible or acceptable to the patient.

Additionally, the optimal treatment parameters for rESWT remain to be determined. Variables such as energy intensity, number of shots, frequency of sessions, and treatment intervals have not yet been optimized. Further research should specifically address these factors to improve therapeutic outcomes and reduce both financial and logistical burdens on patients and caregivers.

In conclusion, while ESWT has shown potential as an intervention for spasticity, its efficacy in HSP remains to be fully established. In this case, functional improvements were observed following rESWT despite unchanged MAS scores, suggesting that the therapy may enhance gait performance through mechanisms other than direct spasticity reduction. The possibility of placebo effects or other confounding factors cannot be excluded, and further research – including randomized, placebo-controlled trials – is warranted to validate these findings and determine the optimal treatment parameters.

## Acknowledgments

We would like to thank Editage (www.editage.jp) for English language editing.

## Author contributions

**Investigation:** Risa Harada, Ryoga Kashima, Wataru Saho.

**Project administration:** Risa Harada.

**Supervision:** Yoshitada Sakai.

**Writing – original draft:** Akira Shutoh.

**Writing – review & editing:** Risa Harada, Ryoga Kashima, Wataru Saho, Ryo Yoshikawa, Yoshitada Sakai.
